# A Rare Association Between Herpes Simplex Virus Type 1 and Miller-Fisher Syndrome

**DOI:** 10.7759/cureus.38163

**Published:** 2023-04-26

**Authors:** Jared J Bies, Mariam Hassan, Swathi Prakash, Mateo Porres-Aguilar, Diego P Peralta

**Affiliations:** 1 Internal Medicine, Texas Tech University Health Sciences Center El Paso, El Paso, USA; 2 Infectious Diseases, Texas Tech University Health Sciences Center El Paso, El Paso, USA

**Keywords:** campylobacter jejuni, areflexia, ophthalmoparesis, ataxia, anti-gq1b ganglioside, miller fisher syndrome, herpes simplex virus 1

## Abstract

The etiopathogenesis for Guillain-Barré syndrome (GBS) and Miller-Fisher syndrome (MFS), a variant of GBS, is well-documented in the literature. However, the association between MFS and an underlying herpes simplex virus type 1 (HSV-1) infection is very limited. We present a unique case of a 48-year-old man who developed diplopia, bilateral ptosis, and gait instability following an acute diarrheal illness and recurring cold sores. The patient was diagnosed with MFS precipitated by recurrent HSV-1 infection following a *Campylobacter jejuni* acute infection. The diagnosis of MFS was supported by a positive anti-GQ1b ganglioside immunoglobulin (Ig)G and abnormal MRI-enhancing lesions of the bilateral cranial nerves III and VI. Intravenous immunoglobulin and acyclovir produced a significant clinical response in the patient within the first 72 hours. Our case highlights the rare association between two pathogens and MFS and the importance of recognizing risk factors, symptomatology, and appropriate workup accompanying an atypical MFS case.

## Introduction

Miller-Fisher syndrome (MFS) is an acute, immune-mediated demyelinating polyneuropathy and a rare Guillain-Barré syndrome (GBS) variant. It was first diagnosed by Miller Fisher in 1956 [1]. MFS is characterized by the infamous triad, ataxia, ophthalmoparesis, and areflexia, following a bacterial or viral infection [2-5]. The commonly implicated pathogens include *Campylobacter jejuni*, cytomegalovirus, Epstein-Barr virus, varicella-zoster virus, and human immunodeficiency virus. These pathogens may elicit an aberrant autoimmune response with measurable anti-GQ1b ganglioside antibodies in serum [6,7]. MFS is less commonly reported after *Mycoplasma pneumoniae* infection or in combination with other autoimmune conditions [8,9]. Herpes simplex virus type 1 (HSV-1) is associated with axonal degeneration and demyelination coinciding with the presentation of GBS or its variant MFS. However, MFS and HSV-1 association is seldom reported in accessible literature [2-9]. Moreover, the association of MFS with two or more pathogens is rare. Therefore, we report a novel MFS case precipitated and/or exacerbated by the co-infection of HSV-1 and *C. jejuni*.

This article was previously presented as a meeting abstract poster presentation at the 2023 South Regional Meeting on February 2, 2023, at the InterContinental New Orleans in New Orleans, Louisiana. 

## Case presentation

A 48-year-old man without significant medical history presented with diplopia, bilateral ptosis, and gait instability. Over a four-day period before evaluation, the patient experienced acute horizontal painless diplopia worsening to oblique diplopia. Then, he developed left upper eyelid ptosis, progressing to bilateral ptosis followed by gait instability. Upon further questioning, the patient stated that two weeks prior to his presentation, he had a non-bloody diarrheal illness for seven days that self-resolved. Cold sores developed on his lips a few days before the diarrheal illness. The patient initially presented to urgent care with these symptoms and was referred to our hospital for further assessment. On presentation to the hospital, the patient was vitally stable. The examination revealed left-sided ptosis with a positive ice test and limited right eye abduction with a positive curtain sign. Initial laboratory workup showed the following (Table 1) results.

**Table 1:** Initial laboratory workup results

Tests	Normal Range	Results
White blood count	4.5-11.0 x 10^3^/μL	9.49 x 10^3^/μL
Red blood count	3.5-5.5 x 10^6^/μL	6.21 x 10^6^/μL
Hemoglobin	12.0-15.0 g/dL	17.0 g/dL
Platelets	150-450 x 10^3^/μL	295 x 10^3^/μL
Sodium, serum	135-145 mmol/L	140 mmol/L
Potassium, serum	3.5-5.1 mmol/L	4.3 mmol/L
Chloride, serum	98-107 mmol/L	110 mmol/L
Bicarbonate	22-30 mmol/L	20 mmol/L
Glucose	74-106 mg/dL	115 mg/dL
Blood urea nitrogen, serum	7-17 mg/dL	16 mg/dL
Thyroid-stimulating hormone	0.465-4.680 mIU/L	1.560 mIU/L
Glycated hemoglobin A1c	< 5.7 %	5.8 %
HIV ½ Rapid 4th Generation	Non-reactive	Non-reactive

An electrocardiogram showed sinus bradycardia with sinus arrhythmia. A transthoracic echocardiogram displayed a normal-sized left ventricle with mildly reduced systolic function. The ejection fraction estimate was 45-50% with grade I diastolic dysfunction. The right ventricle was mildly dilated, the right ventricular systolic function was normal, and there was trace mitral regurgitation.

Initial MRI head with contrast showed no abnormalities. Cerebrospinal fluid (CSF) analysis disclosed an albuminocytological dissociation with a normal white blood cell count, absent red blood cells, elevated protein level, and normal glucose level. HSV-1 was detected in the CSF using BioFire (BioFire Diagnostics, Utah, United States) (Table 2).

**Table 2:** Cerebrospinal fluid analysis

	Reference Range	Results
Opening pressure	10-20 cm H_2_O	N/A
Color	-	Colorless
Appearance	-	Clear
Red blood cells	<5/µL	Absent
White blood cells	<5/µL	2/μL
Glucose	40-70 mg/dL	59 mg/dL
Protein	12-60 mg/dL	65 mg/dL
Herpes simplex virus 1	Negative	Positive

**Figure 1:**
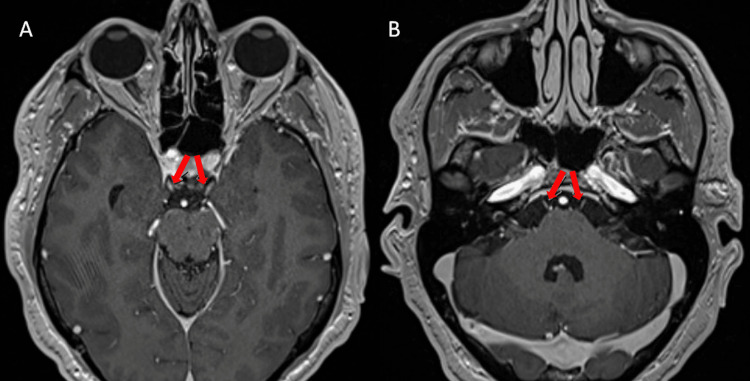
MRI head with contrast images MRI head with contrast shows enhancement of cisternal segments of bilateral cranial nerves III (A) and VI (B)

The patient was started on intravenous immunoglobulin (IVIg) for MFS and acyclovir for HSV-1. Clinical improvement was noted within the first 72 hours with the resolution of his diplopia, bilateral ptosis, and gait instability. A repeat MRI head with contrast was significant for enhancing cisternal segments of bilateral cranial nerves III and VI (Figure 1). *C. jejuni *serum antibody and stool antigen were positive, and so was anti-GQ1b ganglioside IgG, supporting the diagnosis of MFS.

## Discussion

GBS and MFS are not individual entities but rather comprise a continuous spectrum of pathology through the etiopathogenesis of autoantibody formation against gangliosides via molecular mimicry of pathogen-borne antigens [2,6,10,11]. The specificity of these autoantibodies, anti-GQ1b, contributed to the patient's clinical presentation [10]. The subtype of GBS and syndrome severity are also partly determined by the nature of the antecedent infection [12]. These antibodies, however, are not only seen in MFS but also in its complete forms, such as acute ophthalmoparesis, acute oropharyngeal palsy, acute ataxic neuropathy, and Bickerstaff brainstem encephalitis, confirming the broad clinical spectrum associated with these autoantibodies [13,14]. The broad clinical spectrum and manifestations associated with these antibodies are shown in Table 3.

**Table 3:** Cases of MFS and associated clinical manifestations MFS: Miller-Fisher syndrome; URI: Upper respiratory infection; IVIg: Intravenous immunoglobulin; LMWH: Low-molecular-weight heparin; IgG: Immunoglobulin G; CN: Cranial neuropathy; CSF: Cerebrospinal fluid; HSV-1: Herpes simplex virus type 1

Reference, year	Age (years)	Sex	Symptom Onset	Treatment Course	Symptom Resolution	Complications	Positive Serology
Bushra JS, 2000 [1]	11	M	Five days post right ear pain	IVIg	Four months	None	Anti-Mycoplasma pneumoniae IgG
Teener JW, 2013 [4]	49	M	Two weeks post mild diarrhea	Symptomatic care only	Six months	None	Anti-GQ1b antibodies
Al Othman et al, 2019 [3]	28	F	Six days of decreased visual acuity without preceding event	Prednisolone, chloroquine, and LMWH	One month after onset	None	GD1a, GT1a, Gq1b, Lupus anticoagulant
Hsueh et al., 2004 [9]	6	F	Following Mycoplasma pneumoniae infection	N/A	N/A	N/A	N/A
Yuki et al., 2000 [10]	Seven patients	Not provided	Variable	Variable	Variable	Variable	Anti-GQ1b IgG
De Bruyn et al., 2019 [13]	Eight patients	5 M, 3 F	Variable	7 had URI, 1 had gastroenteritis	Mean = 2.5 months	None	Anti-GQ1b antibodies
Odaka et al., 2001 [14]	194 patients (median age = 37)	106 men, 88 women	Symptoms post URI, diarrhea	Variable	Variable	Variable	Anti-GQ1b IgG
Garcia-Rivera et al., 2001 [15]	57	M	No preceding event	Symptomatic	N/A	None	MRI enhancement of bilateral 3^rd^, 6^th^, and 7^th^ CN.
Hattori et al., 1999 [16]	25	F	Post URI	Plasmapheresis x6, IVIg	N/A	N/A	IgG anti-GQ1b and GD1a antibodies
Kiphuth et al., 2009 [17]	23	M	N/A	Immunosuppressive treatment	N/A	N/A	MRI revealed enhancement of CN and cauda equina
Dilena et al., 2016 [18]	Six months	M	Two days post rhinitis	IVIg, plasmapheresis	Six months	Tracheostomy	CSF positive for HSV-1, negative for anti-Gq1b

Although our patient recently experienced a diarrheal illness which contributed towards the formation of anti-GQ1b ganglioside IgG autoantibodies via molecular mimicry, HSV-1 is likely to have played a role in the onset or exacerbation of an already developing MFS picture. We propose further research to establish a direct association between HSV-1 and MFS.

MRI of the brain with gadolinium is an excellent confirmatory test for diagnosing MFS in the correct clinical setting [15]. MFS-affected patients show pathologic gadolinium-enhancing lesions in cranial nerves, specifically the bilateral oculomotor, facial, and abducens nerves, and the cauda equina [16,17]. Our patient had bilateral oculomotor and abducens cranial nerve enhancement, supporting the MFS diagnosis.

The treatment of GBS and its variants comprises IVIg and plasma exchange therapy, with majority experiencing a gradual recovery [19]. The treatment strategy for MFS remains the same irrespective of the underlying infectious cause. In a rare and devastating case of fulminant infantile GBS variant presenting as a peripheral locked-in syndrome and associated with HSV-1, the progressive improvement could be appreciated when the patient was started on acyclovir, IVIg and plasmapheresis [18]. Nevertheless, there remains a paucity of literature that directly correlates the potential of HSV-1 to precipitate or expedite the process of developing MFS.

## Conclusions

Although MFS is self-limited and void of significant neurologic sequelae, it can be incredibly distressing for patients. Clinicians are encouraged to well-acquaint themselves with the risk factors, symptomatology, and appropriate workup that should accompany this acute condition. Many acute infections can lead to the formation of autoantibodies towards gangliosides and subsequently manifest as GBS or its subtypes, such as MFS, in this patient. Given the non-specific nature of anti-GQ1b ganglioside IgG autoantibodies, HSV-1 infection may further provoke the development of these autoantibodies, albeit unusual, and lead to the presentation described above.
